# Transcriptome analysis of *xa5*-mediated resistance to bacterial leaf streak in rice (*Oryza sativa* L.)

**DOI:** 10.1038/s41598-020-74515-w

**Published:** 2020-11-10

**Authors:** Xiaofang Xie, Zhiwei Chen, Binghui Zhang, Huazhong Guan, Yan Zheng, Tao Lan, Jing Zhang, Mingyue Qin, Weiren Wu

**Affiliations:** 1 College of Life Sciences, Fujian Agriculture and Forestry University, Fuzhou, China; 2 Fujian Key Laboratory of Crop Breeding by Design, Fujian Agriculture and Forestry University, Fuzhou, China; 3 Key Laboratory for Genetics, Breeding and Multiple Utilization of Crops, Ministry of Education, Fujian Agriculture and Forestry University, Fuzhou, China; 4 Institute of Tobacco Science, Fujian Provincial Tobacco Company, Fuzhou, China

**Keywords:** Genetics, Genomics, Transcriptomics

## Abstract

Bacterial leaf steak (BLS) caused by *Xanthomonas oryzae* pv. oryzicola (*Xoc*) is a devastating disease in rice production. The resistance to BLS in rice is a quantitatively inherited trait, of which the molecular mechanism is still unclear. It has been proved that *xa5*, a recessive bacterial blast resistance gene, is the most possible candidate gene of the QTL *qBlsr5a* for BLS resistance. To study the molecular mechanism of *xa5* function in BLS resistance, we created transgenic lines with RNAi of *Xa5* (LOC_Os05g01710) and used RNA-seq to analyze the transcriptomes of a *Xa5*-RNAi line and the wild-type line at 9 h after inoculation with *Xoc*, with the mock inoculation as control. We found that *Xa5*-RNAi could (1) increase the resistance to BLS as expected from *xa5*; (2) alter (mainly up-regulate) the expression of hundreds of genes, most of which were related to disease resistance; and (3) greatly enhance the response of thousands of genes to *Xoc* infection, especially of the genes involved in cell death pathways. The results suggest that *xa5* is the cause of BLS-resistance of QTL *qBlsr5a* and it displays BLS resistance effect probably mainly because of the enhanced response of the cell death-related genes to *Xoc* infection.

Bacterial leaf streak (BLS) is a disease caused by the gram-negative bacterial pathogen *Xanthomonas oryzae* pv. oryzicola (*Xoc*) in rice. BLS is one of the most devastating quarantine diseases^1^ in the main rice-producing areas of the world and can cause significant yield loss. BLS resistance is quantitatively inherited in rice^2^. In contrast to qualitative disease resistance, which is controlled by major resistance (R) genes, race-specific, and easily defeated by co-evolving pathogens^3^, quantitative disease resistance is driven by multiple genes, generally non-race-specific and much more durable than qualitative resistance^4–7^. Therefore, breeding of disease-resistant cultivars is an effective strategy to control BLS. However, such effort has often been compromised due to lack of understanding of the underlying molecular mechanism for BLS resistance.

To date, at least 13 quantitative trait loci (QTLs) conferring BLS resistance have been mapped in rice^2,8,9^. In addition, a recessive gene *bls1* showing race-specific resistance to BLS^10^ and a locus *Xo1* conferring complete resistance to the African clade of *Xoc* strains of BLS^11^ are also reported. Interestingly, a non-host resistance gene *Rxo1* from maize also displays qualitative resistance to BLS in rice^12^, which specifically activates multiple defense pathways related to hypersensitive response (HR) against *Xoc*, including some signaling pathways and basal defensive pathways such as the ethylene (ET) and salicylic acid (SA) pathways^13^.

Overexpression of *Differentially Expressed Protein Gene 1* (*DEPG1*), a resistance gene that contains a nucleotide-binding site-leucine rich repeat (NBS-LRR) domain, results in increased susceptibility to *Xoc* strain RS105 and inhibition of some genes related to basal defensive pathways, implying its role of negative regulation for immunity in rice^14^. Similarly, suppression of some defense related (DR) genes, such as *OsWRKY45-1*^15^, *OsMPK6*^16^ and *NRRB*^17^, also presents negative regulation for immunity, increasing resistance to BLS. On the contrary, overexpression of genes *OsPGIP4, GH3-2*, and *OsHSP18.0-CI* significantly enhances resistance to BLS in rice, implying their positive regulation roles for resistance to *Xoc*^18–20^.

In our laboratory, a major QTL *qBlsr5a* conferring BLS resistance was previously mapped on rice chromosome 5^2^ and further fine mapped to a 30-kb interval^21^. Three genes were annotated in the interval. Among them, LOC_Os05g01710 was considered to be the most possible candidate gene, which encodes a transcription initiation factor IIA gamma (TFIIAγ) protein. There were two nucleotides different between the LOC_Os05g01710 allele from the resistant parent and that from the susceptible parents, resulting in a substitution at the 39th amino acid between their encoding proteins^21^. Interestingly, the allele of LOC_Os05g01710 from the resistant parent was identical to *xa5* in sequence, a recessive resistance gene against bacterial blight caused by *Xanthomonas oryzae* pv. oryzae (*Xoo*)^22,23^, implying that suppression of the dominant allele of LOC_Os05g01710 (*Xa5*) from the susceptible parent would increase the BLS resistance if it is really the gene responsible for the *qBlsr5a* effect. This implication has been verified by the result of RNA interference (RNAi) of *Xa5*^21,24^, in which the *Xa5*-RNAi lines display enhanced resistance to BLS with little influence on agronomic traits (only slightly reducing plant height)^24^. Therefore, it is rational to infer that *xa5* is the cause of the BLS-resistance effect of *qBlsr5a*, although the possibility of contribution from the other two genes in the interval cannot be completely excluded.

The present study aimed to investigate the molecular mechanism of *xa5*-mediated resistance to BLS in rice by analyzing the effects of *Xa5*-RNAi on gene expression regulation and transcriptional response to BLS pathogen *Xoc*. We revealed that *Xa5*-RNAi could greatly alter (mainly up-regulate) the expression of many genes related to disease resistance and enhance transcriptional response to *Xoc*, and *Xa5* affects BLS resistance probably mainly by regulating the expression of genes involved in cell death.

## Results

### *Xa5*-RNAi plants and their BLS resistance

**Figure. 1:**
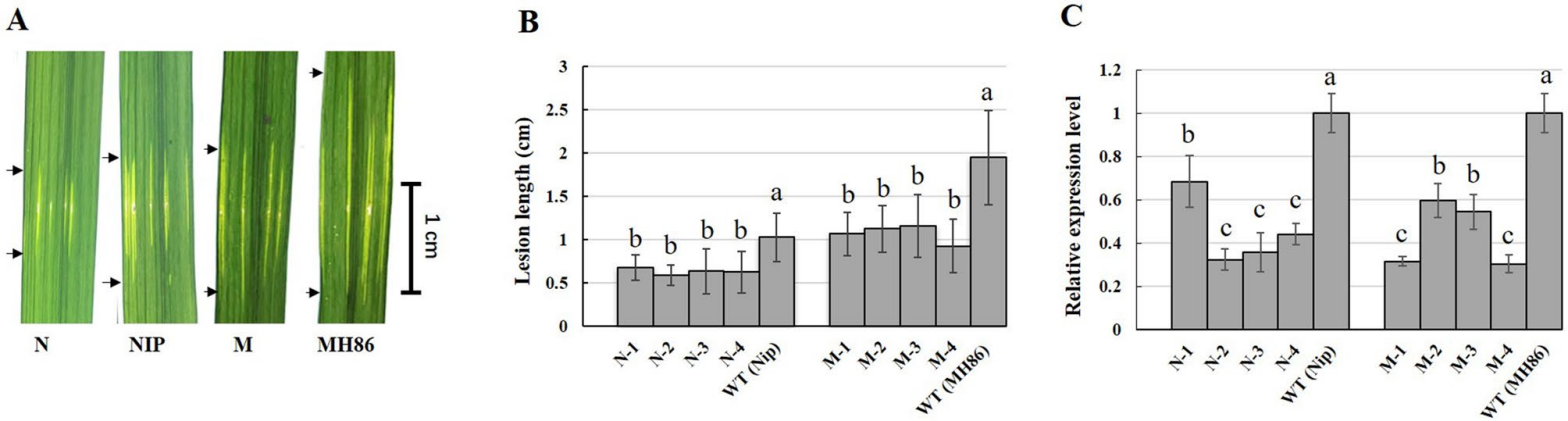
Performance of BLS resistance in *Xa5*-RNAi lines from Nipponbare and MH86. (**A**) Leaves of WT (NIP and MH86) and RNAi lines with BLS lesions at 15 days after *Xoc* inoculation, N and M represent RNAi lines derived from Nipponbare and MH86, respectively. (**B**) Lesion lengths of WT and T_2_ RNAi lines measured at 15 days after *Xoc* inoculation. (**C**) Relative expression levels of *Xa5* in WT and T_2_ RNAi lines at 15 days after *Xoc* inoculation. RNAi lines of N-1/2/3/4 and M-1/2/3/4 were derived from Nipponbare and MH86, respectively. Letters indicate the statistical significance of difference at 0.01 level according to ANOVA-Tukey’s test.

In total, 13 and 9 independent transgenic (T_0_) *Xa5*-RNAi (abbreviated as XR) plants were obtained from rice cultivars Nipponbare and Minghui 86 (MH86), respectively, and 8 T_2_ XR lines were subsequently derived, with 4 from Nipponbare (N-1/2/3/4) and MH86 (M-1/2/3/4) each. Inoculation test showed that the mean lesion length in the XR lines was significantly decreased in comparison with that in wild-type (WT) plants (Fig. 1A,B). qRT-PCR analysis indicated that *Xa5* expression was significantly reduced in the XR lines (Fig. 1C). The lesion length and the *Xa5* expression level were significantly correlated, with a correlation coefficient of 0.93 (*P* < 0.05) in the Nipponbare XR lines and 0.95 (*P* < 0.05) in the MH86 XR lines, respectively. These results reconfirmed that *xa5* can enhance BLS resistance, and suggested again that *xa5* is the cause of BLS-resistance of QTL *qBlsr5a*.

### Reads of RNA sequencing

Since the XR line M-4 from MH86 showed the most significant suppression of *Xa5* expression and increase of BLS resistance (Fig. 1), it was used for RNA-seq together with MH86. In total, 12 RNA samples were sequenced, of which one sample was discarded due to poor sequencing quality. Therefore, a total of 11 RNA samples were analyzed (Table 1). More than 50 million reads were obtained in each sample, of which most (> 84%) were uniquely mapped to the Nipponbare reference genome (Table 1). These uniquely mapped reads were used for subsequent gene expression analysis. The RNA-seq data have been submitted to the database of the NCBI Sequence Read Archive (https://trace.ncbi.nlm.nih.gov/Traces/sra) under the accession number PRJNA558068.

**Table 1:** Statistics of RNA-seq reads.

Samples	Total reads	Uniquely mapped reads	Multiply mapped reads
XRI-1	59,395,708	50,385,642 (84.83%)	1,834,255 (3.09%)
XRI-2	71,452,986	60,635,913 (84.86%)	1,366,235 (1.91%)
XRM-1	54,530,318	47,518,520 (87.14%)	922,469 (1.69%)
XRM-2	59,370,030	52,035,796 (87.65%)	1,343,567 (2.26%)
XRM-3	62,654,504	52,749,774 (84.19%)	1,346,979 (2.15%)
WTI-1	52,052,314	45,482,023 (87.38%)	1,326,379 (2.55%)
WTI-2	60,142,368	52,009,381 (86.48%)	1,723,060 (2.86%)
WTI-3	58,057,590	50,885,173 (87.65%)	1,148,390 (1.98%)
WTM-1	57,207,506	49,886,919 (87.20%)	1,121,745 (1.96%)
WTM-2	51,845,292	45,544,161 (87.85%)	1,018,135 (1.96%)
WTM-3	53,018,932	46,897,761 (88.45%)	891,019 (1.68%)

Reads were mapped to the Nipponbare reference genome.

*XRI* M-4 inocula, *XRM* M-4 mock-inocluated, *WTI* MH86 inoculated, *WTM* MH86 mock-inoculated.

**Figure. 2:**
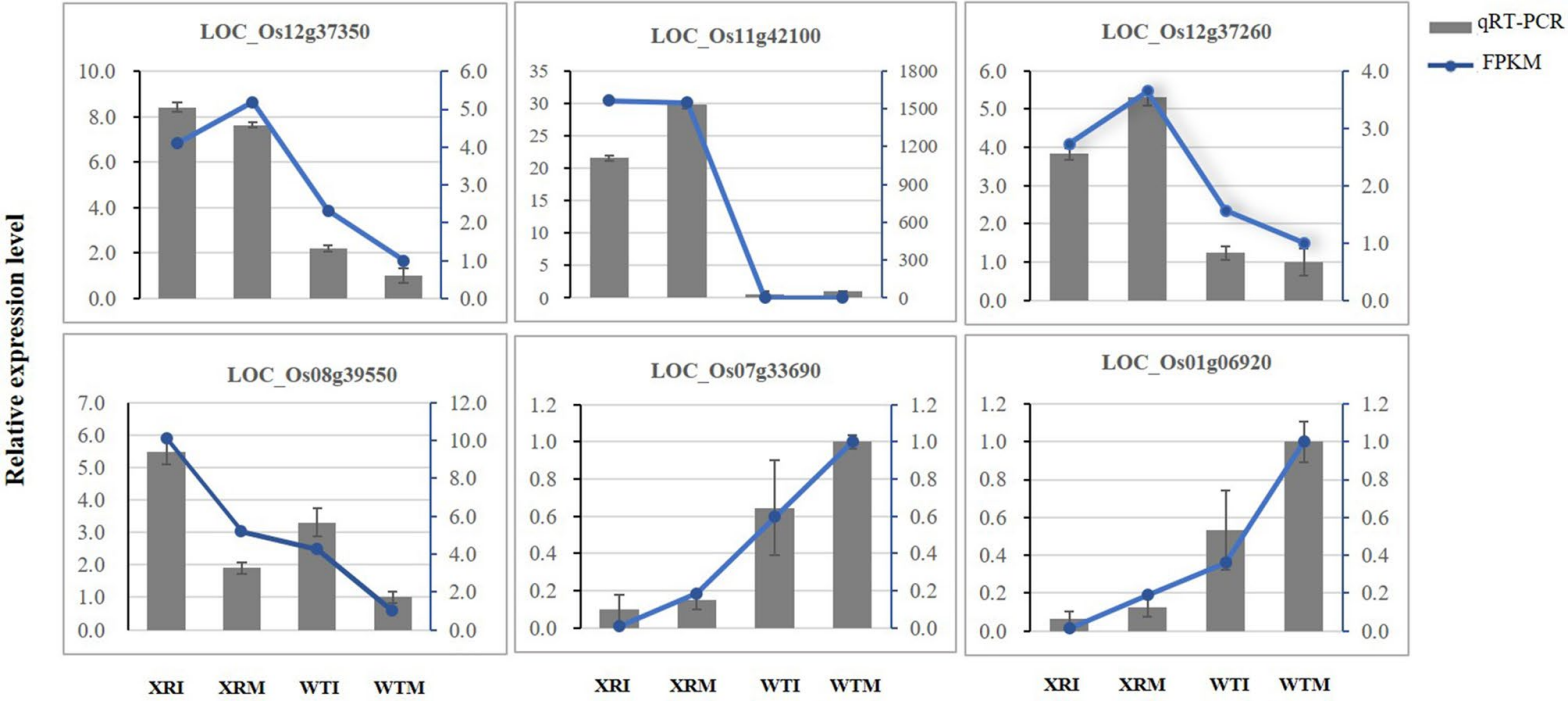
Quantitative RT-PCR analysis of six genes to validate the RNA-seq data.

To validate the RNA-seq data, qRT-PCR (Supplementary Table S1) was used to examine the expression of 6 genes encoding proteins related to disease resistance (including phytohormones, phyto-oxygenase, etc.) in the four treatments. The expression patterns of these six genes detected by qRT-PCR were consistent with those detected by RNA-seq (Fig. 2), indicating that the RNA-seq data were reliable.

### Differentially expressed genes

We analyzed differentially expressed genes (DEGs) in four pairs of comparison, namely, C1: XRM vs. WTM; C2: WTI vs. WTM; C3: XRI vs. XRM; and C4: XRI vs. WTI. C1 and C4 were used to examine the effect of XR on gene expression under normal condition and under the stress of *Xoc* infection, respectively, while C2 and C3 were used to examine the influence of XR on transcriptional response to *Xoc* infection. The four comparison combinations together would show the interaction between XR and *Xoc* infection on gene expression, which would help us understand the molecular mechanism underlying the BLS resistance mediated by *Xa5*.

A total of 317 DEGs were detected in C1 (Supplementary Table S2), among which ~ 3/4 (244) genes were up-regulated, suggesting that *Xa5* functions mainly as a negative regulator for many genes. To examine whether, how many and what kinds of genes related to the resistance to biotic stress existed among the DEGs detected in C1, an overview of regulation and biotic stress showing the transcriptional changes basing on the software of MapMan was generated. MapMan analysis showed that many of the up-regulated genes were involved in the biological pathways related to biotic stress (Supplementary Fig. S1), including hormone signaling, cell wall, beta glucanase, proteolysis, defense genes (DR), redox state, signaling, Myb, secondary metabolites, etc. Most of these genes, such as those associated with cell wall function and phytohormones (salicylic acid, jasmonic acid and ethylene), have been found to play important roles in plant defenses against pathogens^25^, including *Xoc* in rice^16,17,19^.

**Figure. 3:**
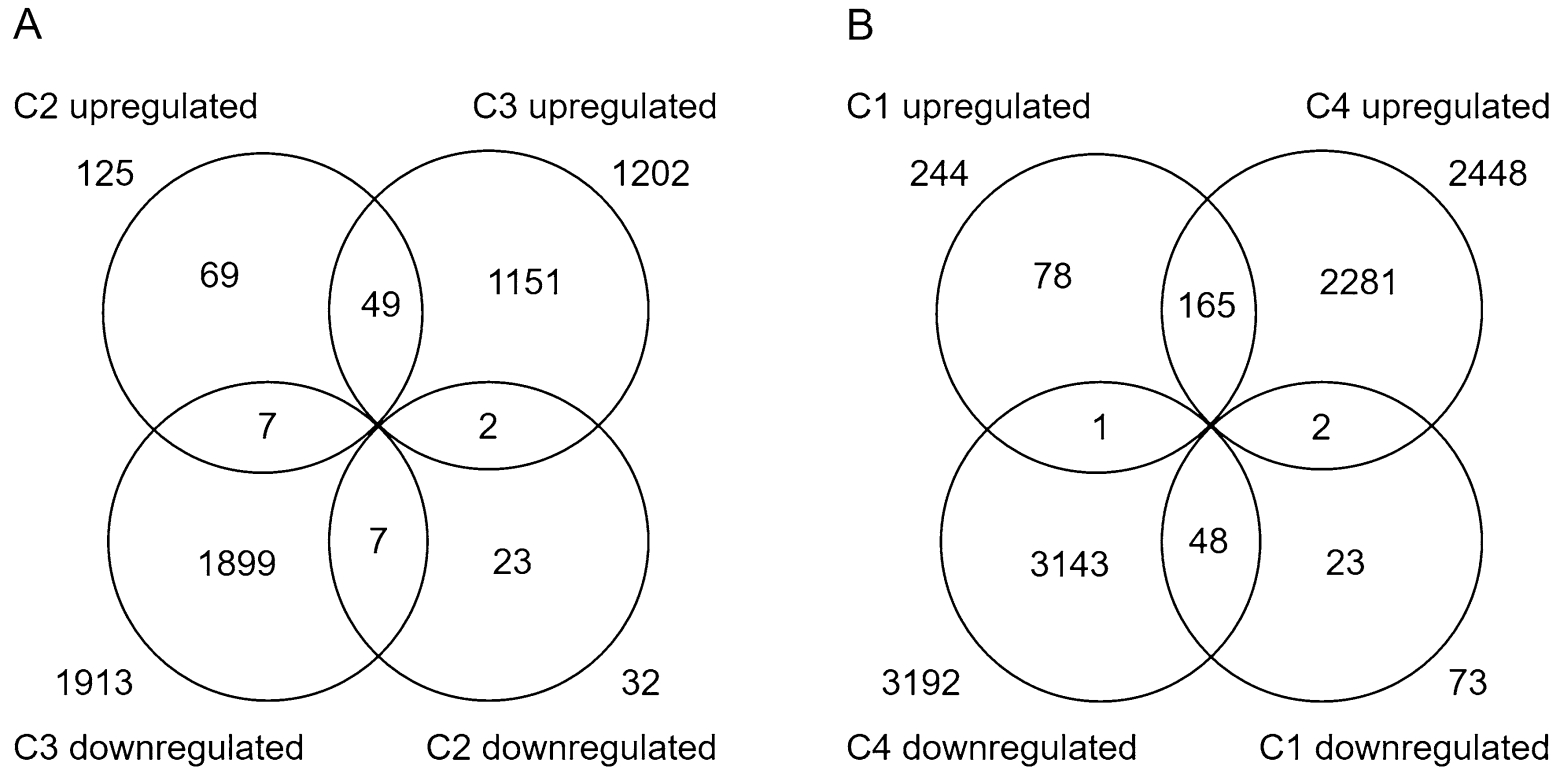
Venn diagram of DEGs detected by RNA-seq in C2 (WTI vs. WTM) and C3 (XRI vs. XRM) (**A**) and that in C1 (XRM vs. WTM) and C4 (XRI vs. WTI) (**B**).

There were 157 DEGs (125 up-regulated + 32 down-regulated) in C2 (Supplementary Table S3) and 3115 DEGs (1202 up-regulated + 1913 down-regulated) in C3 (Supplementary Table S4), respectively. The number of DEGs in C3 was ~ 19 times more than that in C2, suggesting that XR can dramatically enhance the transcriptional response to *Xoc* infection. However, the set of DEGs in C3 only covered ~ 1/3 (56/157) of that in C2, although most of the overlapped DEGs showed the same expression change directions in C2 and C3 (Fig. 3A). In addition, while there were more up-regulated genes than down-regulated genes in WT (C2), the trend was reversed in XR (C3; Fig. 3A). These results suggested that XR can also significantly alter the pattern of transcriptional response to *Xoc* infection.

The number of DEGs identified in C4 was the highest, up to 5640 (2448 up-regulated + 3192 down-regulated; Supplementary Table S5), which was ~ 18 times as many as that in C1 (Fig. 3B). About 2/3 of the DEGs in C1 also existed in C4, displaying consistent expression change directions with only a few exceptions (Fig. 3B). These results indicated that *Xoc* infection greatly augmented the difference of gene expression profile between XR and WT.

### Gene ontology enrichment

Gene ontology (GO) analysis showed that the DEGs in C1 were significantly enriched (*P*-value < 0.01) in 43 GO terms (Supplementary Table S6), including 29 terms on biological process (BP), 1 on cellular component (CC) and 13 on molecular function (MF). Among the BP terms, ~ 3/4 (22/29) were known to be related to plant disease resistance, which could be classified into several groups, including oxidation-reduction^26^, siderophore^27^, secondary metabolite^28^, cell death^29^ and so on. This was consistent with the result of MapMan analysis, suggesting that a main role of *Xa5* is to regulate the expression of genes related to disease resistance.

In C2, 78 GO terms were significantly enriched (*P*-value < 0.01) with DEGs (Supplementary Table S7), including 41 on BP, 9 on CC and 28 on MF, respectively. Almost all of the BP terms were related to disease resistance, which could also be classified into several groups similar to those observed in C1 except for the group of cell death.

The DEGs in C3 were enriched (*P*-value < 0.01) in 66 GO terms (Supplementary Table S8), including 18 terms on BP, 21 on CC and 27 on MF, respectively. Among the BP terms, eight were related to plant disease resistance, including three about cell death, one about defense response, and four about thiamine metabolism. Thiamine is known to function as an activator of plant disease resistance^30^. The three terms about cell death were the most significant, with *P*-values (≤ 1.1 × 10^−11^) at least 10^5^ times smaller than any other term, implying that this group of terms is particularly important for the function of *Xa5* on BLS resistance.

**Figure. 4:**
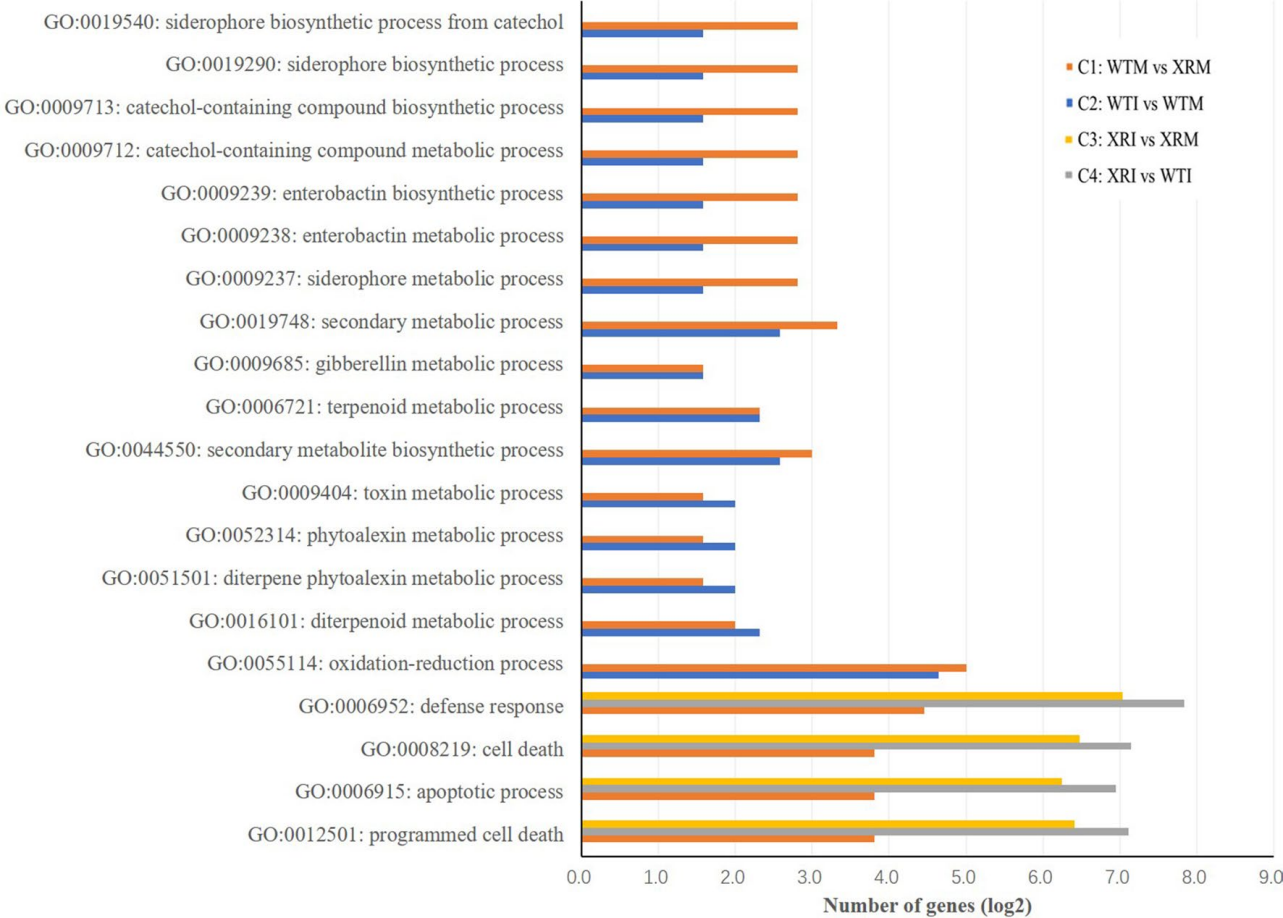
Significant GO terms on biological process common in at least two of the four pairs of comparison.

Surprisingly, although there were much more DEGs in C3 than in C2, the number of enriched GO terms on BP in C3 was much smaller than that in C2. Comparison showed that there were no common BP terms between C2 and C3. However, they had 16 and 4 BP terms common with C1, respectively, among which the common terms between C1 and C2 were all about the basal defense processes, while those between C1 and C3 were about cell death only (Fig. 4).

C4 had 79 GO terms enriched (*P*-value < 0.01) with DEGs (Supplementary Table S9), including 33 on BP, 13 on CC and 33 on MF, respectively. Among the BP terms, nine were related to plant disease resistance, on the aspects of defense response (1 term), cell death (3 terms), immune (2 terms), and others. Similar to the case of C3, C4 had no BP terms common with C2, but four (the one about defense response and the three about cell death) common with C1, which were also common with C3 (Fig. 4). Besides, there was an additional BP term about photosynthesis common between C3 and C4. Noticeably, the three terms about cell death were also the most significant among all BP terms in C4, with *P*-values (≤ 5.8 × 10^−15^) at least 10^5^ times smaller than any other term, implying again the special importance of the genes related to cell death in the *Xa5*-mediated BLS resistance.

## Discussion

TFIIAγ is a component of the general transcription factor IIA and is involved in all polymerase II-dependent transcription in eukaryotes^31,32^. Two TFIIAγ-like genes, TFIIAγ1 and TFIIAγ5 (*Xa5*), have been found in rice. The recessive allele *xa5*, which encodes a V39E substitution variant of TFIIAγ5, is functionally confirmed to enhance the resistance to rice bacterial blight^22,23^. In addition, the results of fine mapping of QTL *qBlsr5a*^21^ and RNAi of *Xa5*^21,24^ have suggested that *xa5* is the most possible candidate gene of the QTL *qBlsr5a* conferring resistance to BLS. This study verified the *Xa5*-RNAi effect on BLS resistance (Fig. 1), further affirming the inference that *Xa5* is responsible for the effect of *qBlsr5a* on BLS resistance.

It is generally considered that plants have evolved two different innate immune systems: the pathogen-associated molecular pattern (PAMP)-triggered immunity (PTI) system and the effector-triggered immunity (ETI) system^33^. PTI belongs to a relatively weak immune response triggered by PAMPs, which depends on the basal defense to restrict the colonization of invading pathogens^34^. As PTI has no race specificity, it is predicted to confer durable and broad spectrum resistance^35^. ETI is mediated by polymorphic resistance proteins that can recognize the highly variable effectors from pathogens, with a fast and strong response usually accompanied with a hypersensitivity reaction (HR) and eventually programmed cell death (PCD) to restrict biotrophic cellular pathogens^36^. We have seen above that the DEGs in C1 were mainly enriched in four groups of GO terms on BP related to disease resistance, namely, oxidation–reduction, siderophore, secondary metabolite, and cell death (Supplementary Table S6). Among these GO terms, the former three groups belong to the mechanisms of basal defense, while the last group (cell death) belongs to the mechanism of HR. Therefore, they are responsible for PTI and ETI, respectively. This suggests that *Xa5* regulates both PTI- and ETI-related genes.

It is interesting that some of the GO terms related to disease resistance in C1 were also detected in C2, C3 and C4 (Fig. 4). Among them, the PTI-related terms were only detected in C2, while the ETI-related terms were only detected in C3 and C4, and there was no overlap between C2 and C3 and between C3 and C4. In addition, the ETI-related terms were very highly significant and also the most significant in both C3 and C4 (Supplementary Tables S8, S9). These results indicated that the ETI-related terms became significant only under the *Xa5*-RNAi condition, no matter whether there was *Xoc* infection (in C3 and C4) or not (in C1), but *Xoc* infection could greatly increase the number of DEGs in these GO terms (Fig. 4). Hence, it is likely that the *xa5*-mediated BLS resistance is mainly due to the response of the genes involved in the cell death pathways to *Xoc* infection. It appears that the dominant allele *Xa5* can inhibit the response of these genes to *Xoc* infection. Therefore, no GO terms about cell death could be detected in C2.

Certainly, PTI-related genes may also contribute to disease resistance. But why the above-mentioned PTI-related GO terms were not significant in C3 and C4? The possible reason could be that the response of the genes in these PTI-related GO terms to *Xoc* infection is similar to that to *Xa5*-RNAi (Fig. 4), and the effects of these two types of response are not additive but superimposed. Thus, since the response of the genes to *Xa5*-RNAi has already existed in an XR line, the response to *Xoc* infection is masked and therefore becomes undetectable.

Taken together, we have seen in this study that as a component of a general transcriptional factor, *Xa5* plays an important role in the regulation of many genes related to disease resistance (including both PTI-related and ETI-related genes) in rice. Suppression of its expression can lead to defense-oriented reprogramming and thereby limit the multiplication or spread of the BLS pathogen *Xoc* through a stronger and more direct immune response like ETI to protect the plant. This defense strategy could accompany with a similar occurrence of a hypersensitivity reaction and eventually result in programmed cell death, cell death, or apoptosis to against the pathogen invasion, although the BLS resistance in rice is controlled by multiple genes and *Xa5* functions only as a QTL.

It has been mentioned above that the recessive allele *xa5* confers qualitative resistance to bacterial blight caused by *Xoo*^22,23^. Therefore, *Xa5* has much stronger effect on bacterial blight resistance than that on BLS resistance. It is found that *Xoc* and *Xoo* induce almost completely different transcriptional changes in the host^37^, implying that *xa5* might mediate different pathways to defend against *Xoo* and *Xoc*. However, considering that cell death is usually a result from hypersensitivity reaction occurring in qualitative resistance, we have reason to expect that the possible *Xa5*-mediated cell death mechanism for BLS resistance found in this study might probably also play a role in bacterial blight resistance.

## Conclusions

The recessive gene *xa5* is the cause of BLS resistance of QTL *qBlsr5a*. It displays BLS resistance effect probably mainly due to the enhanced response of the cell death-related genes to *Xoc* infection.

## Material and methods

### Plant materials

An *indica* rice cultivar Minhui 86 (MH86), which was created and released by Sanming Academy of Agricultural Sciences, Fujian, China, and a *japonica* rice cultivar Nipponbare, which is the cultivar used in the International Rice Genome Sequencing Project, were used in this experiment. Both cultivars were susceptible to BLS.

### Vector construction and rice transformation

**Figure. 5:**
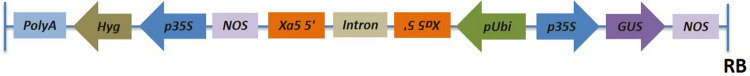
Schematic diagram of *Xa5-*RNAi construct.

To construct *Xa5*-RNAi (abbreviated as XR) vector, a 515-bp fragment containing the whole coding sequence of LOC_Os05g01710 (*Xa5*) was amplified from the cDNA of Nipponbare by PCR. The amplified fragment was inserted into the pTCK303 vector downstream of the ubiquitin promoter, in both forward and reversed orientations (Fig. 5). The vector was introduced into *Agrobacterium* strain EHA105 using the freeze–thaw method, and further into the calli of Nipponbare and MH86 derived from mature embryos following the protocol of Ref.^38^. Positive transgenic lines were identified by PCR using the hygromycin specific primers. Ten positive T_2_ plants from each line were selected for quantitative real-time PCR (qRT-PCR) analysis and BLS-resistance assessment. All the primers used for XR vector construction and transgenic line identification are shown in Table 2.

**Table 2:** Primers used in vector construction and transgenic plant identification.

Application	Primer name	Primer Sequence (5′–3′)^a^
RNAi template	R-*Kpn* I-F	GGGGTACCTCTGGAATTTGCTCGCGTTC
	R-*BamH* I-R	CGGGATCCATACAGCTCTCAGGAAGCCC
	R-*Spe* I-F	GGACTAGTTCTGGAATTTGCTCGCGTTC
	R-*Xba* I-R	GCTCTAGAAAACCCTGACCTCGCAGTTA
Hygromycin	Hyg-R	ACGGTGTCGTCCATCACAGTTTGCC
Identification	Hyg-F	TTCCGGAAGTGCTTGACATTGGGGA

^a^Restriction site sequences are underlined.

### Pathogen infection and resistance assessment

The *Xoc* strain used for inoculation was kindly provided by Prof. Guoying Cheng of Huazhong Agricultural University. Rice plants were inoculated using the pricking inoculation method^2^ at the active tillering stage and lesion length was scored from three leaves of each plant 15 days after inoculation. The resistance ability of a line was indicated by the mean lesion length of 10 plants.

### Quantitative real-time PCR analysis

Total RNA of leaves was extracted using TRIzol reagent (Invitrogen, https://www.invitrogen.com). First-strand cDNA synthesis was performed using PrimeScript RT Reagent Kit with gDNA Eraser (Takara, Japan) following the manufacturer’s instruction. The cDNA samples were then assayed by qRT-PCR using SYBR Premix Ex Taq (Takara). The gene-specific primers used for qRT-PCR analysis are listed in Supplementary information. *Actin* was used as an internal control. Two or more biological replicates and three technical replicates were tested. The relative expression level of a gene was calculated using the 2^−ΔΔCt^ method^39^. The paired t-test method was used to examine the difference of gene expression level between different samples.

### RNA sequencing and data analysis

Leaves of wild-type (WT) MH86 and one of its XR lines (M-4) at the active tillering stage were inoculated with *Xoc* or sterile water (mock inoculation, as control) and total RNA was extracted from the leaves 9 h after inoculation, according to the findings that the expression levels of genes related to BLS resistance drastically change in the time interval of 6–12 h after inoculation^24,37^. In total, there were four treatments: MH86 mock (denoted as WTM), MH86 inoculated (WTI), M-4 mock (XRM), and M-4 inoculated (XRI). Three biological replicates were set for each treatment. Therefore, there were 12 RNA samples in total. Each RNA sample was from a mixture of three 1-cm leaf segments next to the inoculation site. The RNA samples were sequenced on Illumina HiSeq 2000 performed by Biomarker Technologies (https://www.biomarker.com.cn/, BioMarker, Beijing, China). Reads (100 bp in length, paired-end) were mapped to the reference genome and genes available at the Rice Genome Annotation Project (https://rice.plantbiology.msu.edu). For gene expression analysis, the numbers of matched reads were normalized by the RPKM (reads per kb per million mapped reads) method^40^. DEGs were identified using the criteria of |log_2_(fold change)|≥ 2 and false discovery rate (FDR) ≤ 0.01. Gene ontology (GO) analysis was performed based on the Gene Ontology Database (https://www.geneontology.org/). The MapMan tool (https://MapMan.gabipd.org) was used for a graphical overview of pathways involving the DEGs.

## Supplementary information

Supplementary Information 1.

Supplementary Information 2.
