# Supplementary material for: Transcriptome analysis of xa5-mediated resistance to bacterial leaf streak in rice (Oryza sativa L.)
Source: Sci Rep. 2020 Nov 10;10:19439. doi: 10.1038/s41598-020-74515-w (PMC7656458; doi:10.1038/s41598-020-74515-w)
Supplement: Supplementary file 2 — Supplementary Information 2. [file 41598_2020_74515_MOESM2_ESM.docx]

**Transcriptome analysis of *xa5*-mediated resistance to bacterial leaf streak in rice (*Oryza sativa* L.)**

Xiaofang Xie^1,2,3^, Zhiwei Chen^2,3^, Binghui Zhang^4^, Huazhong Guan^2,3^, Yan Zheng^1,2,3^, Tao Lan^2,3^, Jing Zhang^2,3^, Mingyue Qin^1^, Weiren Wu^2,3*^

^1^ College of Life Sciences, Fujian Agriculture and Forestry University, Fuzhou, China

^2^ Fujian Key Laboratory of Crop Breeding by Design, Fujian Agriculture and Forestry University, Fuzhou, China

^3^ Key Laboratory for Genetics, Breeding and Multiple Utilization of Crops, Ministry of Education, Fujian Agriculture and Forestry University, Fuzhou, China

^4^ Institute of Tobacco Science, Fujian Provincial Tobacco Company, Fuzhou, China

^*^ Corresponding author, E-mail: wuwr@fafu.edu.cn

**Supplemental Table S1** Primers used for qRT-PCR in this study

| Gene ID | Name | Primer Sequence (5′-3′) |
| --- | --- | --- |
| LOC_Os03g50885 | OsActin1F | GATTGCCAAGGCTGAGTACGA |
|  | OsActin1R | AAAAGAAGAAACAAGCAGGAGGA |
| LOC_Os05g01710 | Xa5-qRT-2F | ATGACGGAAGCCTTGGAGAA |
|  | Xa5-qRT-2R | CACAGGCCACAATCTTCACC |
| LOC_Os08g39550 | OsPR1-F | GGGTGTTCTCGTCGTGGA |
|  | OsPR1-R | TGTAGCCGGACATGACGC |
| LOC_Os11g42100 | OsPR2 -F | GCCCGACTAGCAACCATCA |
|  | OsPR2 -R | CGCTGGAGGTAGGACATCTC |
| LOC_Os07g33690 | OsPR3-F | CCACCTTTCCCTTGGCAAAA |
|  | OsPR3-R | CGCAACCTCCAAGTCATTCA |
| LOC_Os01g06920 | OsPR4-F | TATTTGAGGTTTCGGGGCTC |
|  | OsPR4-R | TGTTTCCAGTGAAGTTGCTCT |
| LOC_Os12g37350 | OsJA1-F | CTACTTCCCCAACCGTCCC |
|  | OsJA1-R | GCTGGCAAGATGAGGGTAGT |
| LOC_Os12g37260 | OsJA2-F | GACCAAGAACAGACGACCGA |
|  | OsJA2-R | CCGTGTGCATCCCCTCTATA |

**
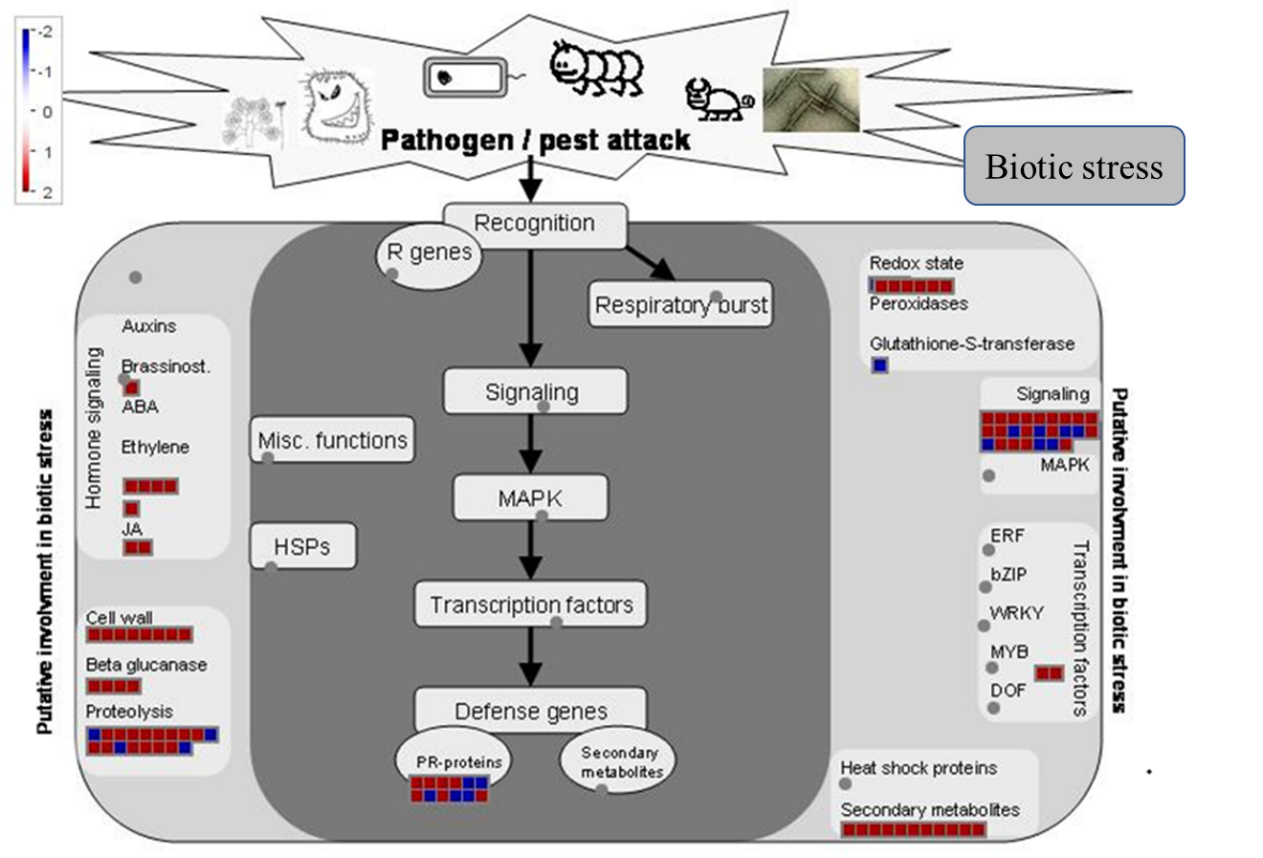
**

**Supplementary Figure S1** MapMan overview of the putative involvement in biotic stress of the transcriptional changes detected in C1 (XRM vs. WTM). Individual genes are represented by small squares, where red and blue indicate significant up-regulation and down-regulation, respectively. The color scale displays log2-transformed fold changes. The results indicate that the RNAi of Xa5 has regulatory effects analogous to those of biotic stress, upregulating the expression of many genes related to plant basal resistance, suggesting that Xa5 is a negative regulator of these genes.
